# Experimental Crack Width Quantification in Reinforced Concrete Using Ultrasound and Coda Wave Interferometry

**DOI:** 10.3390/ma18153684

**Published:** 2025-08-06

**Authors:** Noah Sträter, Felix Clauß, Mark Alexander Ahrens, Peter Mark

**Affiliations:** Institute of Concrete Structures, Ruhr University Bochum, Universitätsstraße 150, 44801 Bochum, Germany; felix.clauss@rub.de (F.C.); alexander.ahrens@rub.de (M.A.A.); peter.mark@rub.de (P.M.)

**Keywords:** ultrasound, coda wave interferometry, reinforced concrete, crack widths, non-destructive testing, strain, fiber optic sensors, digital image correlation

## Abstract

For the first time, comprehensive investigations into the tensile load-bearing behavior and crack formation of reinforced concrete based on ultrasound are presented. Uniaxial tensile tests are performed on reinforced concrete tension members equipped with embedded ultrasonic transducers. Key mechanical parameters across all ranges of tensile behavior are continuously quantified by recording ultrasonic signals and evaluated with coda wave interferometry. The investigations include member configurations of different lengths to cover different numbers of cracks. For reference, crack patterns and crack widths are analyzed using digital image correlation, while the strain in the reinforcement is monitored with distributed fiber optic sensors. For the first time, a direct proportional relationship between the relative velocity change in ultrasonic signals and crack widths is established in the ranges of crack formation and stabilized cracking. In the non-cracked state, linear correlations are found between the velocity change and the average strain, as well as the length of the specimens. The experimental results significantly enhance the general understanding of the phenomena related to ultrasonic signals in flexural reinforced concrete members, particularly concerning cracking in the tensile zone. Consequently, this study contributes to the broader objective of employing coda wave interferometry to evaluate the condition of infrastructure.

## 1. Introduction

The smart combination of the individual strengths of concrete and steel renders reinforced concrete one of the most important building materials today [1]. The utilization of steel reinforcement serves to balance concrete’s deficiency in tensile strength (only about 10% of its compressive strength). Conversely, the concrete ensures corrosion protection for the steel, providing an alkaline environment. Cracking constitutes an intentional and inherent load-bearing principle of reinforced concrete, enabling the redistribution of stresses in the tensile zone to the steel reinforcement in structures subject to bending [2]. The corresponding crack widths are the decisive factor in the durability of the reinforcement and thus of the entire structure. Furthermore, they represent the pivotal element of the design principle “crack before failure”, which guarantees early notification of a potential structural collapse.

Conventional maintenance-related condition assessment usually relies on visual inspection that focuses primarily on crack width [3]. However, due to the labor-intensive nature of the process, these assessments can only be conducted at set intervals and are limited to accessible areas. Non-destructive testing methods overcome these limitations by enabling continuous data collection across entire structures. In the field of reinforced concrete construction, (distributed) fiber optic sensors [4,5,6] have recently emerged alongside conventional strain gauges.

Another option for continuous monitoring of reinforced concrete structures is ultrasound, which enables large areas to be monitored with a few sensors. In the past, coda wave interferometry (CWI) was introduced as a method to determine changes in the velocity of ultrasonic signals, to detect alterations within concrete structures. Unlike conventional methods, which often focus on the time of flight and the initial arrival of ultrasonic signals, CWI analyzes the whole waveform, including the later portions of the signal. Several studies have demonstrated CWI’s sensitivity to small changes in strain and stress [7,8,9,10,11,12,13], as well as to variations in temperature [14,15] and humidity [15,16]. Furthermore, CWI has been successfully employed to localize cracks in reinforced concrete beams [17,18,19,20,21] and tendon breaks in prestressed concrete members [22].

Uniaxial tests are crucial to separately quantify the phenomena of and contributions to ultrasonic signals resulting from tensile or compressive stresses in reinforced concrete. While there have been numerous studies published on combining uniaxial compression tests with ultrasonic measurements (e.g., [23,24,25,26,27,28,29], and specifically in conjunction with CWI, e.g., [9,30,31,32,33]), the same cannot be said for uniaxial tensile tests. To date, only Zhang et al. [34] and Diewald et al. [30] have conducted ultrasonic investigations on uniaxial tensile tests made from plain concrete. However, without reinforcement, these tests are limited to the uncracked, linear-elastic range, which represents only a small portion of the entire load-bearing regime of reinforced concrete structures.

Our research aims to take this a significant step further. For the first time, uniaxial tensile tests on reinforced concrete members integrating ultrasonic measurements are conducted. This approach enables us to quantify the pure tensile load-bearing behavior of reinforced concrete across all stages, from the non-cracked state to crack formation, stabilized cracking, and yielding. Additionally, we establish a direct correlation between the crack width and changes in the ultrasonic velocity, independent of any influence of the compression zone. This makes it possible to directly infer crack widths from the ultrasonic signal and its easily measurable change, even and especially in inaccessible areas. Moreover, we utilize embedded ultrasonic sensors and test configurations of different lengths to derive broad conclusions. For reference, we use distributed fiber optic sensors (DFOS) and digital image correlation (DIC) [35].

The article first recaps the fundamentals of the tensile load-bearing behavior of reinforced concrete and the principles of ultrasonic testing using CWI in Section 2. Section 3 provides details on the testing campaign with reinforced tension members. The basic results are presented in Section 4. Section 5 discusses these and presents the correlation functions, and Section 6 concludes with the main findings.

## 2. Ultrasonic Investigation of Reinforced Concrete Under Tensile Loading

### 2.1. Load-Bearing Behavior of Reinforced Concrete Tension Members

**Figure 1:**
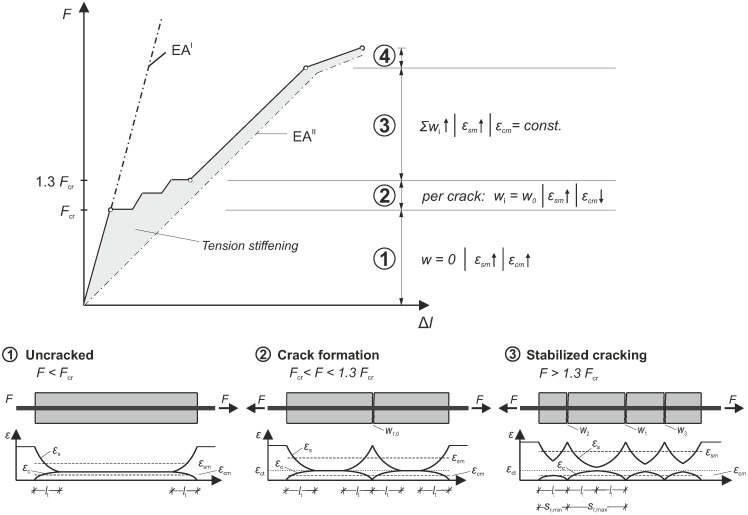
Ranges of the tensile behavior of reinforced concrete using the example of a tension member, adapted from [2].

The load-bearing behavior of reinforced concrete under tensile load (uniaxial tension or tensile zone in bending) is characterized by various ranges. According to [2,36], the tensile behavior is categorized into four main phases: ① uncracked range, ② crack formation, ③ stabilized cracking, and ④ yielding. Figure 1 demonstrates this concept using a tension member, illustrating the load–displacement curve (*F*-Δl–curve) along with corresponding strain curves (concrete strain $\epsilon_{c}$ and steel strain $\epsilon_{s}$) and the associated mechanical quantities. The tension member is a common test setup in the field of reinforced concrete (cf., [37,38,39,40,41]), allowing investigation of tensile load-bearing behavior independently from any compression zones. Initially, the tensile force only acts on the rebar, until it is fully transferred to the concrete through bond stresses.

The uncracked range ① is characterized by a linear-elastic material response. Once the tensile force has been fully introduced into the concrete (via the introduction length $l_{t}$), the strains in the steel rebar and the concrete equalize, and any bond stresses disappear. As the force increases, the steel and concrete strains increase linearly. Although the average steel $\epsilon_{sm}$ and concrete strains $\epsilon_{cm}$ are not identical, they remain proportional to each other as the tensile force increases. In comparison, the uncracked range exhibits the steepest slope of all ranges in the load–displacement diagram as the tensile stiffness EA is at its maximum.

The crack formation range ② starts when the tensile strength of the concrete $f_{ctm}$ (at the corresponding tensile strain $\epsilon_{ct}$) is first reached due to the load increase. At this point, the primary crack of an initial width $w=w_{0}$ suddenly forms, causing the *F*-Δl–curve to shift. At the crack, the tensile force is entirely borne by the rebar. Consequently, the strain in the steel increases, while the strain in the concrete drops to zero.

Away from the crack, the tensile force is reintroduced from the steel to the concrete through bond stresses. This is the same at the extremities of the tension member (the ends can be considered analogous to cracks). The bond length required to completely reintroduce the tensile force can be expressed according to [2,36] using Equation (1), which incorporates the rebar diameter $d_{s}$ and the reinforcement ratio $\rho_{s}$. The mean bond stress $\tau_{sm}$ can be approximated using a factor of 1.8 times the mean tensile strength $f_{ctm}$ [36].

(1) $$l_{t}=\frac{f_{ctm}d_{s}}{\tau_{sm}\rho_{s}}=\frac{d_{s}}{1.8\rho_{s}}\approx 0.555\frac{d_{s}}{\rho_{s}}$$

Once the force has been fully reintroduced, the next crack develops where the concrete’s tensile strength is lowest due to scatter. This process continues as long as the space between two cracks is adequate to completely reintroduce the tensile force into the concrete. According to [2], this process concludes when the load reaches approximately 1.3 times the initial load at cracking.

The stabilized cracking range ③ is reached when all cracks have formed, and the distance between them is no longer adequate to fully reintroduce the tensile force into the concrete. At this point, no additional cracks can form. The spacing between two adjacent cracks $s_{r}$ (or the distance from the outermost crack to the end of the tension member) is defined by a span depending on the length $l_{t}$ necessary to completely reintroduce the tensile force into the concrete.

(2) $$l_{t}=s_{r,min}<s_{r}<s_{r,max}=2l_{t}$$

As the tensile force increases, the existing cracks in the stabilized cracking range widen. While the strain in the reinforcing steel continues to rise (as the rebar still exhibits linear-elastic behavior), the strain in the concrete remains constant. This is due to the fixed length over which the tensile force can be transferred to the concrete. As a result, the strain between the concrete and steel no longer aligns at any point along the tension member.

The constrained strain state of the concrete between the cracks leads to a consistent load-bearing contribution of the concrete. This results in a parallel shift in the *F*-Δl-curve when compared to the purely cracked state, where only the steel bears the load. This load-bearing contribution of the concrete under tensile stress in the cracked state is referred to as tension stiffening.

The final range ④, yielding, starts when the yield strength of the rebar is exceeded. This causes a substantial increase in deformation, leading to large crack widths and significant relative displacements between the concrete and the rebar. As a result, the bond between the concrete and the rebar gradually deteriorates.

### 2.2. Sensing Reinforced Concrete with Ultrasound and Coda Wave Interferometry

Ultrasonic measurements can be used to continuously monitor reinforced concrete structures. This approach exploits the fact that the velocity of ultrasonic signals varies in response to changes in the medium through which they travel. When the medium remains unchanged, the same signals can be consistently received.

Ultrasonic signals are significantly affected by cracks, resulting in a decrease in wave velocity [19,20]. The acoustoelastic effect describes the relationship between the wave velocity and the strain in media [42,43]. An increase in tensile strain reduces the wave velocity. Ultrasonic measurements can therefore effectively capture the key factors influencing the tensile load-bearing behavior of reinforced concrete across its different ranges.

**Figure 2:**
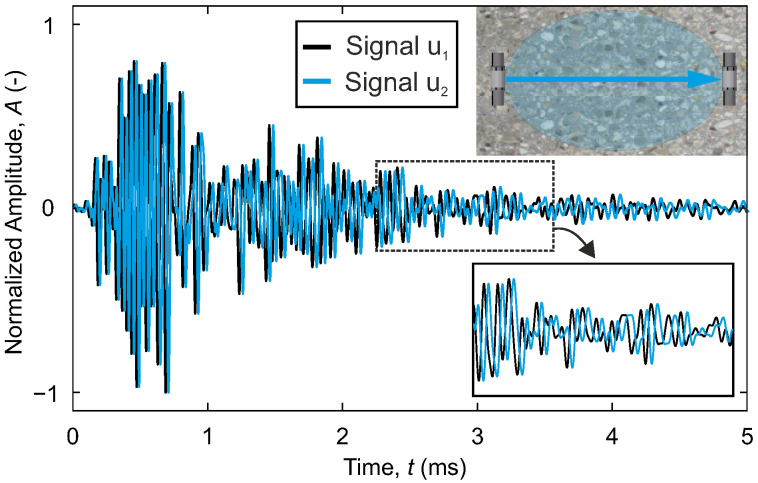
Ultrasonic reference signal $u_{1}$ and shifted signal $u_{2}$ after a change in the medium.

In this article, coda wave interferometry [44,45] is employed to determine velocity changes between the ultrasonic signals. The method involves comparing the waveforms of two signals $u_{1}$ (the reference signal) and $u_{2}$ (the altered signal) within a defined time window ($t_{1}$ to $t_{2}$) using cross-correlation (Figure 2). The cross-correlation coefficient ranges from 1 for identical signals to 0 for completely different signals.

Using the stretching technique [32,46,47], the reference signal $u_{1}$ is stretched by a factor α until the cross-correlation coefficient between the two signals is maximum (Equation (3)). The calculated factor α reflects the negative relative velocity change $\frac{dv}{v}$ [%] between the signals. An increase in the wave velocity of $u_{2}$ compared to $u_{1}$ corresponds to positive values of the relative velocity change, while a decrease is associated with negative values.

(3) $$-\frac{dv}{v}=\underset{\alpha }{arg max}CC\left(t,\;\alpha \right)=\underset{\alpha }{arg max}\frac{\int_{t_{1}}^{t_{2}}u_{1}\left(t\left(1-\alpha \right)\right)\;u_{2}\left(t\right)\;dt}{\sqrt{\int_{t_{1}}^{t_{2}}u_{1}^{2}\left(t\left(1-\alpha \right)\right)\;dt\int_{t_{1}}^{t_{2}}u_{2}^{2}\left(t\right)\;dt}}$$

For continuous repetitive measurements, either a fixed reference can be selected or the reference can be adapted step by step. Stepwise adjustment is recommended for large expected changes (CC<0.7). Then, the incrementally obtained velocity changes between two signals can be accumulated to reset the reference to a fixed point in time. Detailed background information on these techniques is provided in [47].

## 3. Experiments

### 3.1. Experimental Setup

**Figure 3:**
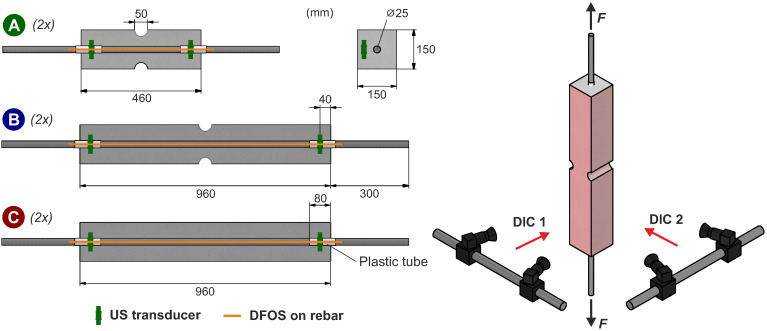
Test setup of specimen configurations A–C and measuring equipment.

**Figure 4:**
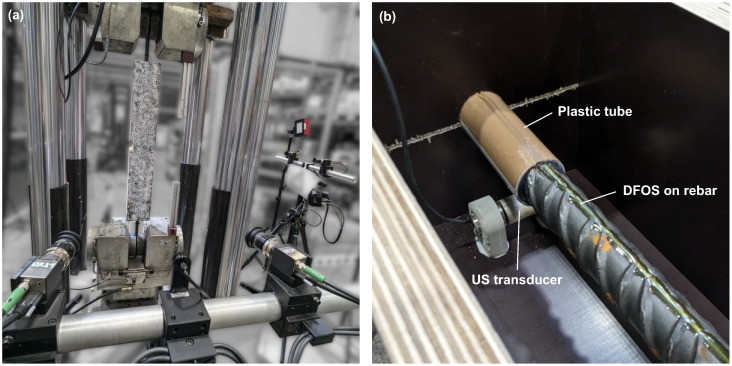
Photo of (**a**) test setup and (**b**) detail of transducer location, plastic tube and FOS on rebar.

To examine the tensile and cracking behavior of reinforced concrete in uniaxial tension with ultrasound, tension members were tested in the laboratory of Ruhr University Bochum, Germany. The setup utilizes concrete prisms (cross-section: 150 mm × 150 mm, mix design acc. to Table 1), through which a steel bar (diameter: 25 mm) runs at barycenter (Figure 3). The bar protrudes at both ends and is pulled by the testing machine (Figure 4a). The test campaign is designed for specimens that exhibit different numbers of cracks and adhere to the following requirements:

- Predefined and easily repeatable crack patterns by specifying the position of the first crack notching the cross-section
- Limitation of the initial crack width $w_{0}$ by selecting a high reinforcement ratio
- Control of the maximum number of cracks in the specimen via its length in relation to the transfer length $l_{t}$

**Table 1:** Concrete mixture of the specimens with aggregate (Agg) sizes.

Cement		w/c	Agg 0/2	Agg 2/4	Agg 4/8	Agg 8/16
	(kg/m^3^)		(kg/m^3^)	(kg/m^3^)	(kg/m^3^)	(kg/m^3^)
CEM I 32.5 R	350	0.60	674	222	410	412

The campaign comprises three different configurations, with two repetitions each. Notched at the center, configuration A is intentionally designed short, to ensure just one crack at the notch, but no additional cracks. In contrast, configuration B is long enough to allow for one additional crack on each side of the notch at the center (3 cracks in total). Configuration C is not notched but has the same length as configuration B. Here, the position of the first crack only depends on the natural scatter of the concrete’s tensile strength. Based on the random position of the first crack, a certain number of further cracks is possible.

### 3.2. Measuring Equipment

Ultrasonic transducers (type S0807 from Acoustic Control Systems, Ltd. (Sarrebruck, Germany)) are embedded at both ends of the specimens. They work as a transmitter and receiver and are firmly glued onto the formwork using specially manufactured cement holders before the concrete is poured (Figure 4b). To prevent any weakening of the concrete cross-section, the specimens are extended by 8 cm at both ends to accommodate the transducers. Plastic tubes around the rebar are used to prevent bonding and to make sure the load transfer starts right after this section.

The transducers are of diameter 20 mm, 75 mm long, and operate at a central frequency of 60 kHz (characterized in [48,49]), rendering them suitable for monitoring cracks in concrete structures [50]. They are connected to a measuring unit that includes a digital-to-analog converter and data acquisition module (USB-6361 from National Instruments (Austin, TX, USA)), an amplifier to amplify the transmission signal, a multiplexer (2701 from Keithley (Thalwil, Switzerland)), and a PC equipped with software (details are provided in [51]).

Digital image correlation [52,53] is used to determine the displacements and the strain field on the concrete surface of the specimens and the crack widths. Two DIC systems (Carl Zeiss GOM Metrology GmbH (Braunschweig, Germany), ARAMIS) in parallel enable the recording of two side faces at once and the capturing of possible misalignment (Figure 3, right). To this end, a speckle pattern of black dots on white is sprayed onto two adjacent surfaces (cf. Figure 4a).

Distributed fiber optic sensors are attached to the rebars. They enable quasi-continuous measurement of the steel strain with a gauge pitch of 0.65 mm. The DFOS is glued into a groove milled into the rebar (1 mm × 2 mm), for which the AC2411 adhesive by Polytec PT (Karlsbad, Germany) is used. This procedure and the measurement setup were examined in detail and published by the authors elsewhere [40,54].

### 3.3. Test Procedure

The tests are conducted as displacement controlled, with the applied tensile force measured by a load cell on the testing machine. The rate is maintained constant at 0.2 mm/min until yielding. Then, the rate is increased to more than 1 mm/min in the yielding range. All measurements (ultrasound, DIC, DFOS) are recorded continuously at a frequency of 0.2 Hz. In the laboratory, temperature and humidity are maintained at constant levels throughout the experiment, to eliminate their potential influence on the ultrasonic and DFOS measurements.

Accompanying tests are carried out in parallel to determine the material parameters of concrete and reinforcement. The mean values from three samples each according to DIN EN 12390 [55] are summarized in Table 2.

## 4. Results

### 4.1. Load, Crack Pattern, and Strain

**Figure 5:**
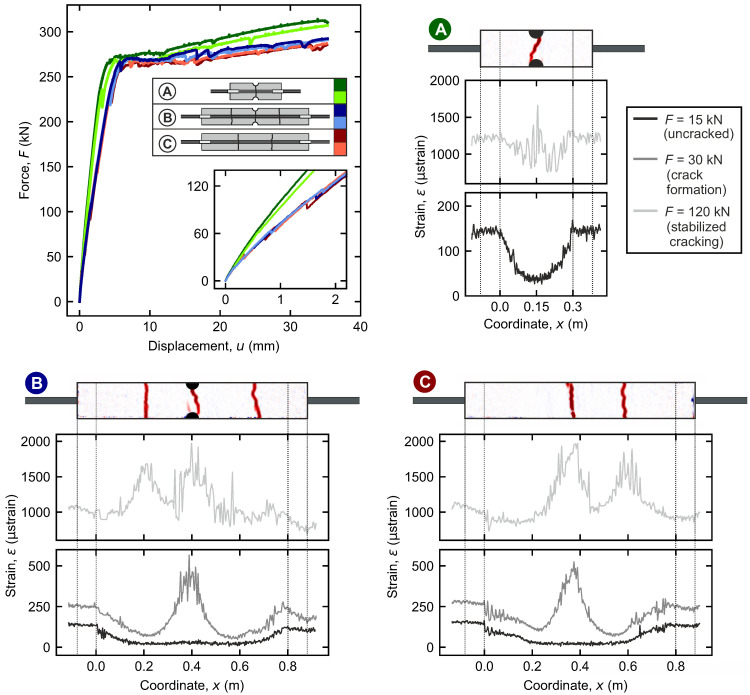
Load–displacement curves for all specimens of the configurations A–C and completed crack patterns from DIC for one specimen of each configuration with corresponding steel strains along the rebar from DFOS.

This section presents the fundamental mechanical results from the tests. At the top left, Figure 5 displays the measured load–deflection curves for configurations A, B, and C (with two specimens each). The configurations are color-coded. In addition, the completed crack patterns from DIC and the corresponding strain curves from DFOS for one specimen of each type at a load level of F=120 kN (light gray) are shown. These are supplemented by the curves for load levels of 15 kN (uncracked, black) and 30 kN (crack formation range, dark gray).

The load–deflection curves are characterized by four sections of different slopes corresponding to the tensile ranges of reinforced concrete introduced above (cf. Section 2.1): linear-elastic, crack formation (indicated by minor drops in the force, cf. zoom in Figure 5), stabilized cracking, and yielding range. Since configurations B and C share the same length, the curves overlap throughout. The shorter configuration A shows slightly steeper branches. Yielding is consistently observed in all configurations at about 260 kN.

Observed using DIC, as expected, configuration A shows a single crack at the notch. The initial crack of configuration B also originates at the notch. Another two cracks form on the sides. Despite the missing notch and general potential for more cracks, both specimens of configuration C show only two cracks.

All cracks coincide with peaks in the strain curves. They indicate locations where the entire tensile force is borne by the reinforcement. In the uncracked range, the strain level is constant in the middle of the specimens, but increases towards the ends due to the load transfer. Recall that in the first 80 mm from both ends of the specimens (cf. Section 3.2), there is intentionally no bond between the reinforcement and the concrete. Here, the force is entirely borne by the rebar and, thus, the strain curve remains constant.

### 4.2. Crack Widths

DIC measurements enable the determination of the crack widths from the distance between two points on the concrete surface (left and right from a crack) using the recorded displacement field. The difference in these distances directly corresponds to the crack width.

**Figure 6:**
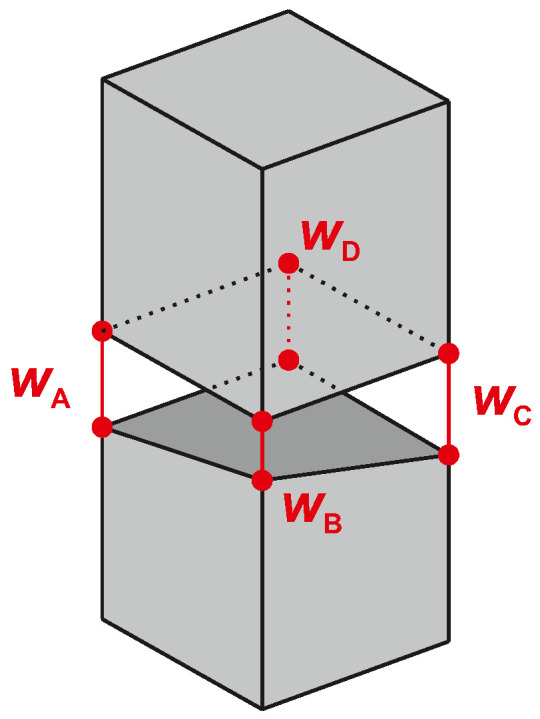
Average, minimum, and maximum crack widths; determination assuming linear misalignment.

Despite all the effort spent on the uniaxial tensile setup, unwanted bending effects arise due to inherent imperfections in both the specimens and the test setup. Consequently, the width of a crack is not uniformly distributed across the specimen. For a deeper analysis, we determine the average crack width $w_{M}$ from the crack surfaces idealized as two inclined planes (see Figure 6). As a result, the crack width changes linearly from the left to the right edges of each side. This is associated with the center of the cross-section and can be calculated as the arithmetic mean of the widths at the opposite edges A–C or D–B:

(4) $$w_{M}=\frac{w_{A}+w_{C}}{2}=\frac{w_{B}+w_{D}}{2}$$

With the crack widths at three edges A, B, and C known, the width of the crack at the missing edge D can be determined.

(5) $$w_{D}=w_{A}+w_{C}-w_{B}$$

Moreover, the minimum and maximum widths of a crack occur at one of the four edges of the specimen:

(6) $$w_{min}=\min\left(w_{A},w_{B},w_{C},w_{D}\right)$$

(7) $$w_{max}=\max\left(w_{A},w_{B},w_{C},w_{D}\right)$$

**Figure 7:**
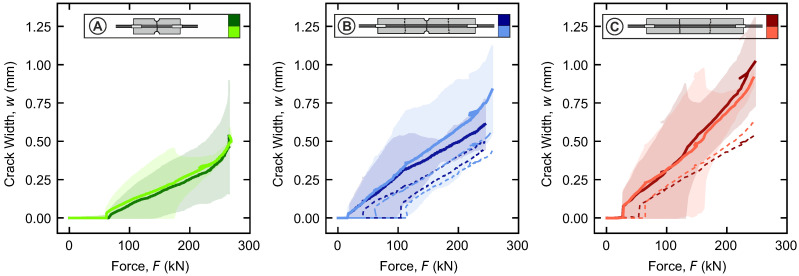
Evolution of crack width with force for specimen configurations A–C—solid lines: averaged width of the initial crack with corresponding ranges between minimum and maximum, dashed lines: averaged widths of the second and third crack.

Two DIC systems in parallel are used to measure the crack width at three edges of all specimens. With the outlined procedure, the average, minimum, and maximum crack widths are determined for the initial cracks, as shown in Figure 7. The average is drawn as a solid line, while the ranges from minimum to maximum are highlighted in the background. Moreover, the evolutions of the average widths for the second and third cracks are drawn as dashed lines.

In all configurations, the width of the initial crack varies significantly between the minimum and maximum (e.g., up to Δw>0.8 mm for C). Despite this variability, the average widths of the initial cracks run consistently in each configuration. They increase almost linearly with the applied force. And this trend also remains the same for the second and third cracks. All cracks form suddenly with an initial width and then grow continuously.

The load at initial cracking also varies between the configurations. Based on the minimum and maximum of the initial crack width, ranges between the initial opening at the first edge and complete opening at the last edge can be identified (A: 65 kN–170 kN, B: 20 kN–100 kN, C: 30 kN–110 kN). These ranges reflect the unwanted bending effects that occur. Possible causes include variations in the concrete tensile strength, as well as geometric imperfections of the specimens and test setup that can lead to misalignment.

Configurations B and C exhibit initial cracking loads that are lower than those calculated (B with notch: $F_{cr,calc}=37\;\mathrm{kN}$, C without notch: $F_{cr,calc}=56\;\mathrm{kN}$). This is attributed to unwanted bending. In contrast, the specimens of configuration A exceed the calculated cracking load. This indicates that, due to their short length, the tensile force could not be fully transferred to the concrete. Thus, a larger load is actually required to exceed the tensile strength. Recall this risk was intentionally taken when designing configuration A. The short length makes sure that only one crack can form at the center.

### 4.3. Ultrasound

**Figure 8:**
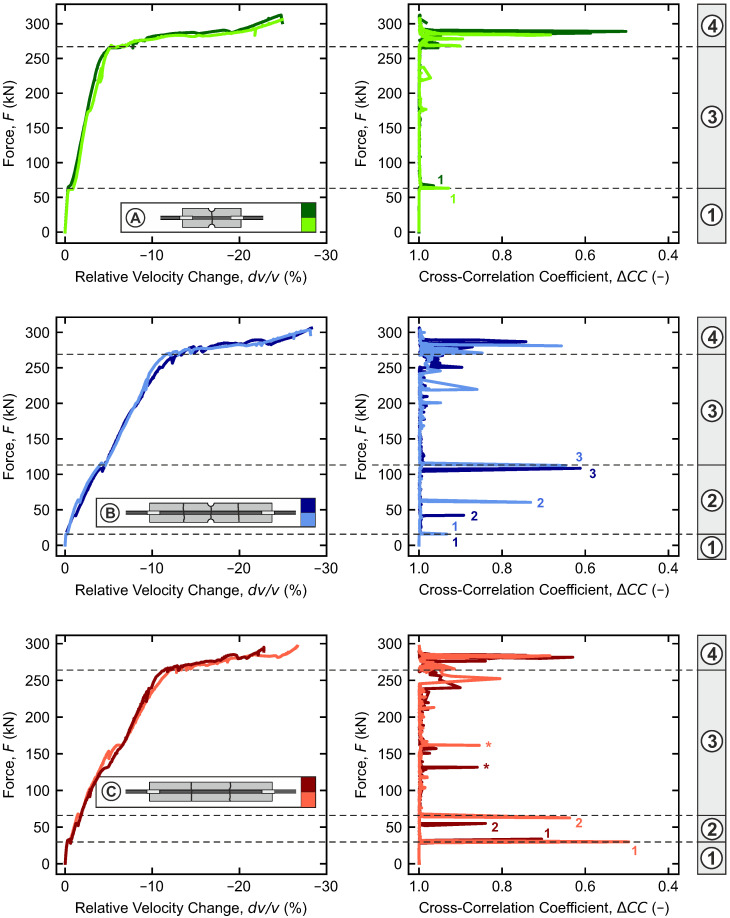
Evolution of relative velocity changes and stepwise cross-correlation coefficients with force for specimen configurations A–C.

Coda wave interferometry is applied to the continuously recorded ultrasonic data using the stepwise method (cf. Section 2). On the left, Figure 8 shows the cumulated relative velocity changes dv/v over the tensile force for all specimens. On the right, the corresponding cross-correlation coefficients ΔCC between the individual signals are presented. Sections of nearly linear slopes can be identified in the courses of the relative velocity changes dv/v. In contrast, all corresponding cross-correlation coefficients remain close to 1. This indicates only minimal changes between the consecutively recorded ultrasonic signals. However, the courses exhibit several localized peaks that can be assigned to the previously identified cracking times (cracks numbered consecutively).

Based on the dv/v-curves and the course of ΔCC, four distinct ranges ①–④ can be identified in the ultrasonic results, which directly correspond to the ranges of tensile behavior described in Section 2 (①: uncracked, ②: crack formation, ③: stabilized cracking, ④: yielding). Overall, the general characteristics of the dv/v-curves are very similar to those of the F−Δl curve.

The change in the relative velocity is smallest in the range ① (uncracked). It always remains below −1%. In addition, peaks are not observed in the course of ΔCC during this phase. At the first crack, marking the end of range ①, the dv/v-curves are shifted and corresponding peaks in ΔCC are observed.

In range ② (further crack formation in configurations B and C), the gradient of the dv/v-curves generally increases. As before, new cracks cause small shifts in dv/v, accompanied by peaks in the course of ΔCC.

In range ③ (stabilized cracking), the slope of dv/v remains the same as in range ②. However, the gradient in configuration A (short specimens) is lower than that in configurations B and C. Over large parts of this range, no shifts or peaks become visible; except for C, where small peaks are caused by the complete opening of the first cracks, as indicated with an * in Figure 8.

The final range ④ (yielding) is characterized by a substantial increase in dv/v, reaching well into the double-digit percentages. Strong peaks in ΔCC occur just before the yielding load is reached at the end of range ③, indicating major changes in the specimens, such as failure of the bond.

## 5. Discussion

This section discusses and quantifies the phenomena affecting the relative velocity changes across the different ranges of tensile behavior of reinforced concrete. The examination covers the uncracked state, crack formation, and the final stabilized cracking range. The yielding range will no longer be considered, as it is not relevant to practical assessments of structures in service conditions.

### 5.1. Uncracked Range

In the uncracked state, the relative velocity change in the ultrasonic signals is affected by increasing tensile strains. To characterize the strain state, the average steel strain $\epsilon_{sm}$ is determined from the DFOS measurements between the ends of the specimens. In the uncracked state, this average steel strain is proportional to the average concrete strain (cf. Section 2.1). Therefore, the average steel strain fully represents the overall strain state.

**Figure 9:**
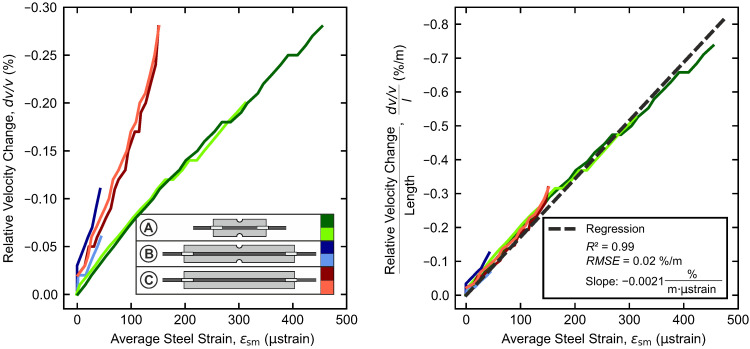
(**Left**): Correlation of relative velocity change and average steel strain for specimen configurations A–C, (**Right**): Normalization by the specimen length.

On the left, Figure 9 directly contrasts the relative velocity change dv/v and the average steel strain $\epsilon_{sm}$ for all specimens, while on the right, the relative velocity changes are normalized by the associated specimen length *l*.

In both figures, clear linear relationships become evident between the change in relative velocity and the average steel strain. The curves for the individual configurations overlap precisely; however, a significantly lower gradient is observed for the short configuration A than for configurations B and C.

Normalization by length accounts for the different slopes and results in aligned curves that all share the same slope. With a high coefficient of determination of $R^{2}=0.99$, the linear regression yields a rate of change of −0.0021%/m·μstrain. In the uncracked range, a clear linear dependency is found between the relative velocity change and the average strain (here, expressed by the average steel strain) in the specimen, as well as the specimen length.

### 5.2. Crack Formation Range

**Figure 10:**
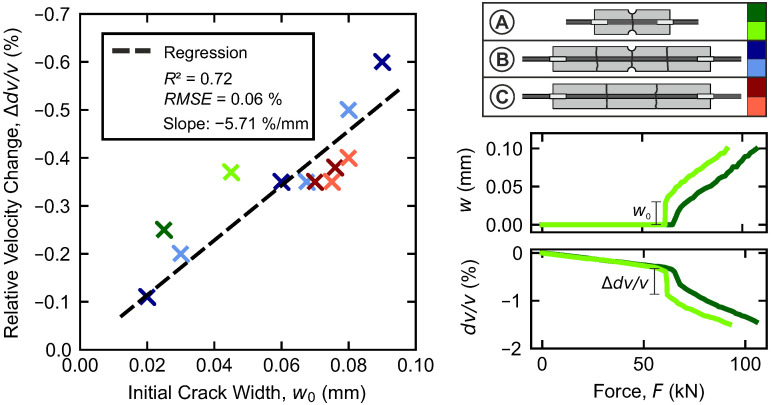
Initial crack width $w_{0}$ plotted against corresponding drop in the relative velocity change for specimen configurations A–C.

In the crack formation range, initial cracking is found to have the main influence on the relative velocity. To quantify the extent, the previously observed shifts (cf. Section 4.3) in the dv/v-curve, which result from suddenly opening cracks, are considered in more detail. Their magnitudes in terms of Δdv/v are compared to the respective initial crack widths $w_{0}$ for all specimens in Figure 10. For this purpose, the average initial crack widths are calculated for all cracks.

In general, the main trend indicates that a greater initial crack width is associated with a greater increase in the relative velocity change. Linear regression yields a rate of −5.71%/mm. However, with an $R^{2}$ value of 0.72, the trend is not particularly well established.

Every crack that forms is linked to a system change, in which some of the tensile force is transferred from the concrete to the reinforcing steel. Besides the impact of crack width on the dv/v-curve, it can be assumed that the resulting changes in strain also affect the course. Given such a complex interplay of different influences, it seems reasonable to conclude that the range of crack formation is much too difficult to quantify accurately using ultrasonic measurements.

### 5.3. Stabilized Cracking

**Figure 11:**
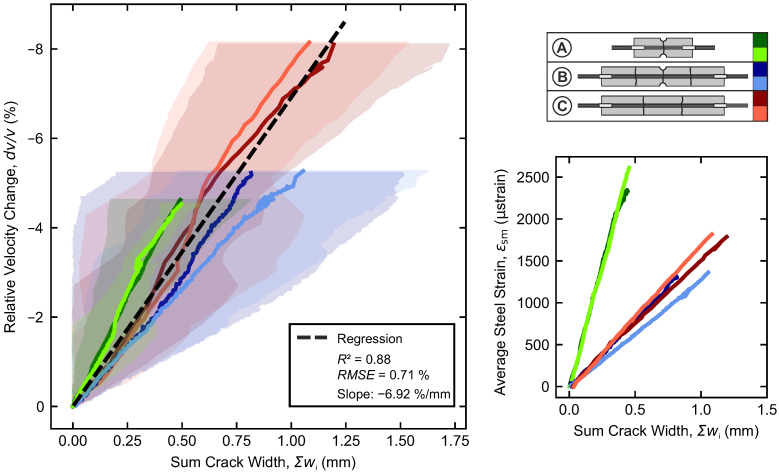
(**Left**): Sum of the crack widths versus relative velocity change, (**Right**): Evolution of the average steel strain in the rebar over the sum of the crack widths.

In the stabilized cracking range, the widths of existing cracks increase, but no additional cracks form. On the left, Figure 11 directly contrasts the crack widths with the relative velocity change in the ultrasonic signals. To ensure comparability among the configurations, the sum of the crack widths Σw (A: one crack, B: three cracks, C: two cracks) between the sender and the receiver is considered here. Solid lines represent the average crack widths, while the corresponding range between the minimum and maximum crack widths is shown in the background.

The averaged accumulated crack widths show an almost linear relationship with the relative velocity change. With a coefficient of determination of $R^{2}=0.88$, linear regression yields a rate of –6.92%/mm. But the curves exhibit significant variation between the minimum and maximum crack widths across all configurations. Nevertheless, the strong correlation demonstrates that individual crack states—characterized by different distributions of widths and numbers of cracks—are effectively represented in the ultrasonic measurements averaged over the sensed area. Obviously, there is no direct dependency on the length in the Σw-dv/v–relationship.

The short configuration A exhibits a slightly steeper slope in the *w*-dv/v–relationship than the other configurations. This difference may be attributed to the small contribution of the steel strains that slightly increase in parallel (Figure 11, right). While the concrete strain remains constant in the stabilized cracking range (cf. Section 2.1), the steel strain continues to rise linearly.

The slope of the average steel strain versus the accumulated crack width is also stronger for the short configuration A than for the others. This phenomenon is due to the influence of the extremities of the specimens, which increases the average steel strain, without any increase in the accumulated crack widths. Due to the shorter length, this is again stronger in configuration A than in the other configurations.

## 6. Conclusions

For the first time, the plain tensile load-bearing behavior of reinforced concrete is fully investigated with ultrasound. For three out of four characteristic ranges, correlation functions are established between the crack width and the relative velocity change in the ultrasonic signal. In practice, they may be used to reliably measure crack widths using ultrasonic techniques, even in inaccessible areas. These functions are experimentally derived from uniaxial tensile tests on RC members with embedded ultrasonic sensors. The individual lengths of the members feature different numbers of cracks. Accompanying measurements with DFOS yield the strains in the reinforcement, while DIC is used to derive the crack width. The results of the ultrasonic measurements are compared and quantified with the mechanical parameters across the different ranges of the tensile load-bearing regime. The following conclusions can be drawn:

- In the uncracked state, the changes in the relative velocity and the average (steel) strain are linearly correlated. A statistically well-determined ($R^{2}=0.99$) length-normalized relationship with a rate of −0.0021%/m·µstrain is established.
- In the crack formation range, each crack causes the relative velocity to drop. In size, the drop is medium-correlated ($R^{2}=0.72$) with the initial crack width, with a rate of –5.71%/mm.
- In the stabilized cracking range, the crack width significantly determines the relative velocity change in the ultrasonic signal. Here, a proportional relationship of dv/v to the sum of the averaged crack widths is established, with a rate of –6.92%/mm ($R^{2}$ = 0.88). This correlation is independent of the specimen length. The ultrasonic signal integrates all cracks, and the distribution of crack widths and feeds, into one quantity dv/v.
- In contrast to the stable states observed in the uncracked and the stabilized cracking ranges, the crack formation range is characterized by system rearrangements, rendering it less quantifiable by ultrasound.

## Figures and Tables

**Table 2:** Material parameters (MPa) from accompanying tests.

$f_{\mathrm{cm},\mathrm{cyl}}$	$f_{\mathrm{cm},\mathrm{cube}}$	$f_{\mathrm{ctm}}$	$E_{\mathrm{cm}}$	$f_{\mathrm{ym}}$
28.4	34.4	2.8	26,200	532

## Data Availability

The original contributions presented in the study are included in the article, further inquiries can be directed to the corresponding author.

## Abbreviations

The following abbreviations are used in this manuscript:

CWI	Coda Wave Interferometry
DFOS	Distributed Fiber Optic Sensor
DIC	Digital Image Correlation
